# European Corn Borer (*Ostrinia nubilalis*) Induced Responses Enhance Susceptibility in Maize

**DOI:** 10.1371/journal.pone.0073394

**Published:** 2013-09-02

**Authors:** Nicole J. Dafoe, James D. Thomas, Paul D. Shirk, Michelle E. Legaspi, Martha M. Vaughan, Alisa Huffaker, Peter E. Teal, Eric A. Schmelz

**Affiliations:** 1 Center of Medical, Agricultural and Veterinary Entomology, Agricultural Research Service, U.S. Department of Agriculture, Gainesville, Florida, United States of America; 2 Department of Molecular Genetics and Microbiology, Center for Neurogenetics, University of Florida, Gainesville, Florida, United States of America; 3 Department of Chemistry, Yale University, New Haven, Connecticut, United States of America; AgroParisTech, France

## Abstract

Herbivore-induced plant responses have been widely described following attack on leaves; however, less attention has been paid to analogous local processes that occur in stems. Early studies of maize (*Zea mays*) responses to stem boring by European corn borer (ECB, *Ostrinia* *nubilalis* ) larvae revealed the presence of inducible acidic diterpenoid phytoalexins, termed kauralexins, and increases in the benzoxazinoid 2-hydroxy-4,7-dimethoxy-1,4-benzoxazin-3-one-glucose (HDMBOA-Glc) after 24 h of herbivory. Despite these rapidly activated defenses, larval growth was not altered in short-term feeding assays. Unexpectedly, ECB growth significantly improved in assays using stem tissue preconditioned by 48 h of larval tunneling. Correspondingly, measures of total soluble protein increased over 2.6-fold in these challenged tissues and were accompanied by elevated levels of sucrose and free linoleic acid. While microarray analyses revealed up-regulation of over 1100 transcripts, fewer individual protein increases were demonstrable. Consistent with induced endoreduplication, both wounding and ECB stem attack resulted in similar significant expansion of the nucleus, nucleolus and levels of extractable DNA from challenged tissues. While many of these responses are triggered by wounding alone, biochemical changes further enhanced in response to ECB may be due to larval secreted effectors. Unlike other Lepidoptera examined, ECB excrete exceedingly high levels of the auxin indole-3-acetic acid (IAA) in their frass which is likely to contact and contaminate the surrounding feeding tunnel. Stem exposure to a metabolically stable auxin, such as 2,4-dichlorophenoxyacetic acid (2,4-D), promoted significant protein accumulation above wounding alone. As a future testable hypothesis, we propose that ECB-associated IAA may function as a candidate herbivore effector promoting the increased nutritional content of maize stems.

## Introduction

Induced defense responses often protect plants against insect herbivory, but in some cases insects modify these responses by manipulating the plant to produce more optimal feeding sites [1–4]. Within 24 h, maize stem feeding by the European corn borer (ECB, *Ostrinia* *nubilalis* ) rapidly induces the local accumulation of defensive compounds such as benzoxazinoids and the kauralexin family of diterpenoid phytoalexins [5]. These defenses do not appear to affect the short-term growth of ECB, which are one of the most devastating pests of maize [5,6]. Larvae typically progress by first feeding on whorl tissue and then tunneling into the stalk where they disrupt vascular transport and facilitate pathogen entry [7]. The long-term accumulation of inducible biochemical defenses has the potential to deter ECB feeding [8]. However, it is also possible that ECB larvae possess mechanisms to overcome defense responses and flourish in nutrient poor tissue.

Currently there are no large-scale studies identifying differentially regulated genes or proteins in maize stems following ECB feeding. In previous foliar studies, the quantities of defensive proteins such as a maize protease inhibitor (MPI) and cysteine protease (MIR1-CP) increased in response to damage caused by various leaf-feeding insects [9–11]. Protease inhibitors (PIs) bind to and inhibit the activity of proteases in insect guts, thereby decreasing the nutritional value of the plant tissue [12]. In maize leaves, MPI is strongly induced at the gene and protein levels in response to wounding and Egyptian cottonworm ( *Spodoptera* *littoralis* ) feeding [10]. Recombinant MPI can effectively inhibit multiple insect gut proteases and when over-expressed in rice results in decreased growth of the striped stem borer ( *Chilo* *suppressalis* ) [10,13]. The protease, MIR1-CP, is also strongly up-regulated in maize whorl tissue in response to fall armyworm ( *Spodoptera* *frugiperda* ) feeding [9,10]. Opposite of MPI, MIR1-CP functions by physically disrupting the larvae’s peritrophic matrix which severely inhibits subsequent growth [14,15].

During herbivory, plants respond to mechanical damage caused by feeding and can additionally react to effectors found in the insect’s oral secretions (OS) [16–18]. Numerous effectors including polypeptides and modified fatty acids that elicit plant defense responses have been identified. For example, a fatty acid-amino acid conjugate, *N*-(17-hydroxylinolenoyl)-L-glutamine termed volicitin, was identified as a potent defense elicitor from beet armyworm ( *Spodoptera* *exigua* ) OS [19]. Similarly, inception-related peptides derived from ATP synthase γ-subunit (cATPC) proteins present in fall armyworm OS activate defenses in cowpea (*Vigna unguiculata*) and the common bean (*Phaseolus vulgaris*) at concentrations as low as 1 fmol leaf^-1^ [20,21]. In contrast, some insect effectors, such as glucose oxidase (GOX) and the cATPC derived peptide Vu-In^-A^, can suppress or antagonize plant defense elicitation [22,23]. Corn earworm ( *Helicoverpa* *zeae* ) larvae secrete GOX while feeding on tobacco (*Nicotiana tabacum*) leaves which in-turn functions to block the wound-induced accumulation of toxic nicotine [22]. Recently *S* *. littoralis* and *Pieris* *brassicae* OS were also found to suppress wound-induced responses in Arabidopsis (*Arabidopsis thaliana*) leading to increased larval growth [3]. To collectively consider these divergent physiological activities, Hogenhout and Bos have recently proposed a broad definition of effector that encompasses “all pathogen/pest proteins and small molecules that alter host-cell structure and function. These alterations may trigger defense responses induced by avirulence factors, elicitors, microbial/pathogen/herbivore-associated molecular patterns or promote infection or both.” [24]. This broad conceptual framework is useful given that closely related biochemicals in naturally occurring elicitor mixtures can also antagonize defense activation [23].

Insect-derived elicitors commonly trigger the synthesis of phytohormones such as jasmonic acid (JA) and ethylene (ET) that subsequently regulate the production of biochemical defenses including volatiles [25,26]. Consistent with this pattern, both JA and ET were produced rapidly in response to ECB feeding and the combination of these hormones differentially regulated benzoxazinoids in maize stem tissue [5]. Several additional phytohormones are also involved in regulating defense responses, including salicylic acid (SA) and indole-3-acetic acid (IAA), both which are generally considered antagonists of JA signaling [27]. SA signaling is associated with protection against biotrophic pathogens while IAA regulates an array of complex roles in development [28,29]. In tobacco ( *Nicotiana* *sylvestris* ), exogenous IAA suppresses the wound-induced production of JA and subsequent accumulation of induced defenses such as nicotine [30]. Upon damage, levels of IAA commonly decrease while JA levels increase [31,32]. Likewise, inhibitors of endogenous auxin transport can result in similar increases in nicotine accumulation [33]. The down-regulation of auxin signaling is an important component of plant resistance to bacteria whereas exogenous application of stable auxin analogs, such as 2,4-dichlorophenoxyacetic acid (2,4-D), increase disease susceptibility [34]. Curiously, in contrast to typical wound or herbivore-induced responses, recent characterization of ECB attack on maize stems demonstrated rapid and sustained accumulation of both JA and IAA in damaged tissues [5].

In young maize leaves, ECB resistance has been strongly correlated to increased levels of the benzoxazinoid, 2,4-dihydroxy-7-methoxy-1,4-benzoxazin-3-one (DIMBOA) [35]. Much less is known about resistance in maize stalks, although it appears to be associated with cell wall composition [35]. Our previous short-term 24 h study demonstrated no inducible defense-related growth inhibition for ECB established in maize stalks [5]. To better understand the molecular and biochemical mechanisms involved in maize stem responses to sustained ECB attack over 48 h, we investigated changes in biochemical defenses, nutritive quality of tissues and how these changes affected further insect growth. We quantified kauralexins, benzoxazinoids, simple carbohydrates, lipids, gene transcript levels, and proteins to determine which are differentially regulated during ECB stem attack. We also detected changes in cellular structure that occur in challenged tissues. While chemical defenses were significantly higher in ECB damaged tissue, larval performance significantly increased. ECB-damaged tissue was more nutritious than control stem tissues, containing greater quantities of proteins, sucrose, and free linoleic acid. To explore mechanisms of how ECB may influence maize stem responses, insect OS and frass contents were chemically analyzed and resulted in the identification of IAA as a major component of these secretions and excretions. Treatment of maize with the metabolically stable synthetic auxin, 2,4-dichlorophenoxyacetic acid (2,4-D), locally elevated total protein levels supporting the hypothesis that ECB-associated IAA may function as a candidate effector promoting the increased the nutritional value of stems.

## Results

### ECB Stem Attack Triggers Maize Responses

**Figure 1:**
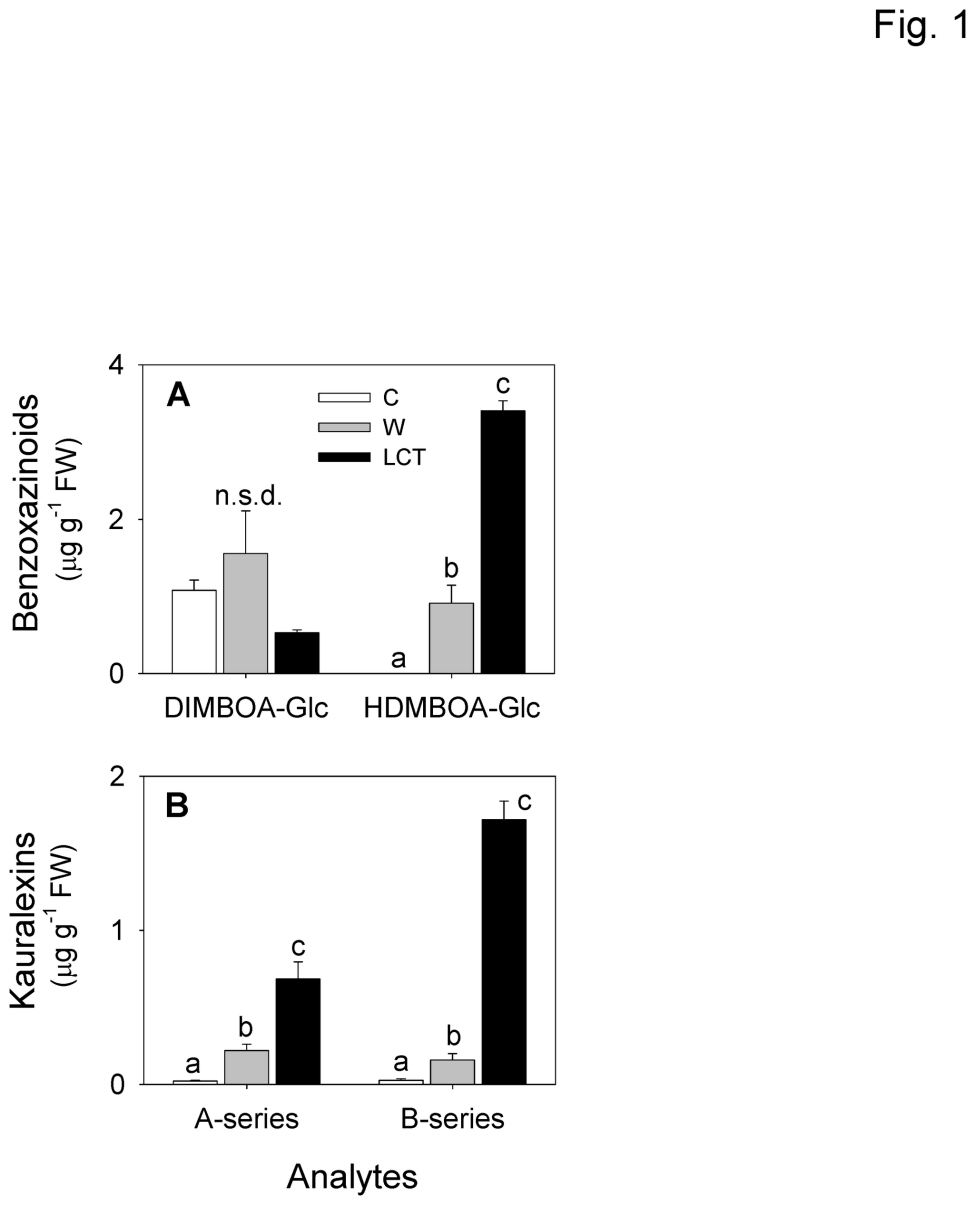
ECB herbivory elicits the accumulation of benzoxazinoids and terpenoid phytoalexins in maize stem tissues. Average quantities (*n* = 3, +SEM) of **A**, 2,4-dihydroxy-7-methoxy-1,4-benzoxazin-3-one)-β-D-glucopyranose (DIMBOA-Glc) and 2-(2-hydroxy-4,7-dimethoxy-1,4-benzoxazin-3-one)-β-D-glucopyranose (HDMBOA-Glc) and **B**, combined totals of kauralexin A and B series diterpenoid phytoalexins in control (white bars), wound (grey bars) and larval-conditioned tissue (LCT, black bars) after 48 h. No significant difference (n.s.d) indicates ANOVA *P* > 0.05. Different letters (a–c) represent significant differences (all ANOVAs *P* < 0.01; Tukey test corrections for multiple comparisons, *P* < 0.05).

To examine if maize defense-related compounds continue to increase with extended periods of herbivory, levels of benzoxazinoids and kauralexins were compared between stem tissues that were left untreated, wounded with a cork borer, or previously ECB-damaged for 48 h. To simplify presentation, we define maize stems damaged by ECB for 48 h as larval-conditioned tissue (LCT). Although there was no significant difference in the levels of DIMBOA-Glc (2,4-dihydroxy-7-methoxy-1,4-benzoxazin-3-one)-β-D-glucopyranose) between control, wound, and LCT treatments, the benzoxazinoid, HDMBOA-Glc (2-(2-hydroxy-4,7-dimethoxy-1,4-benzoxazin-3-one)-β-D-glucopyranose), was detected at significantly elevated levels in wounded tissue and LCT (Figure 1A). While HDMBOA-Glc was undetectable in control tissue, approximately 3.6 µg g^-1^ FW was present in LCT. Moreover, LCT contained 3.7-fold greater HDMBOA-Glc levels than wounded tissue. Total benzoxazinoid concentrations were 3.6-fold and 1.6 fold greater in LCT compared to control and wounded tissue, respectively. HDMBOA is recognized as a highly reactive benzoxazinoid that is not susceptible to detoxification via larval re-glycosylation [36]. Similarly, A- and B-series kauralexins, constituting acidic diterpenoid phytoalexins based on *ent*-kaurane and *ent*-kaur-15-ene hydrocarbon skeletons, respectively, were also significantly increased in LCT (Figure 1B). Total kauralexin concentrations in LCT were 50-fold greater than controls and 6.3-fold higher than wounded tissues. At 48 h, the total levels of both benzoxazinoids and kauralexins exceeded those previously reported at 24 h, which were 1.4 µg g^-1^ FW and 0.23 µg g^-1^ FW respectively [5].

### ECB Herbivory Increases the Nutritional Value of Maize Stem Tissue and Promotes Larval Growth

**Figure 2:**
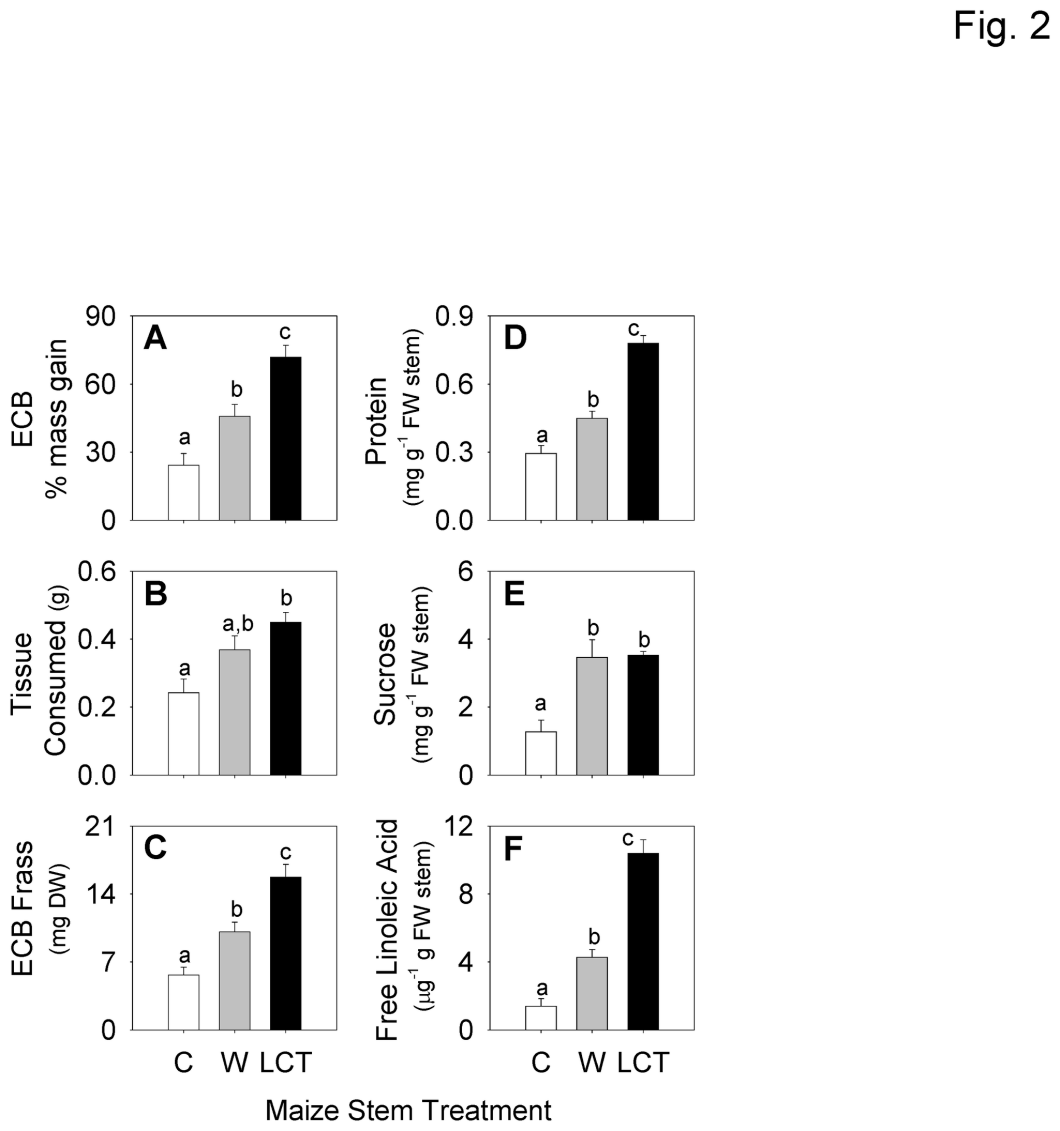
ECB stem herbivory improves host plant quality and subsequent larval growth. **A**, Average (*n* = 11, +SEM) percent mass gain; **B**, tissue consumed and; **C**, frass production for ECB larvae fed for 24 h on stems that were previously treated as control (C), wound (W), or ECB larval-conditioned tissue (LCT) for 48 h. Average (*n* = 6, +SEM) **D**, total soluble stem protein; **E**, sucrose and; **F**, free linoleic acid in comparable 48 h control, wound, and LCT stem tissues. Different letters (a–c) represent significant differences (all ANOVAs *P* < 0.01; Tukey test corrections for multiple comparisons, *P* < 0.05).

To determine if maize defenses induced at 48 h are sufficient to reduce ECB growth, we conducted a preliminary experiment with ECB larvae (previously reared on diet) supplied with control and LCT stems for 24 h. Average (n=18, ±SEM) percent mass gain of larvae on LCT was significantly greater than those supplied with previously untreated control stems (Figure S1A). To better understand the role of mechanical damage alone, the 24 h ECB growth assay (n=11) was repeated using control, wound and LCT tissues. Larval mass increased by 25% after 24 h of feeding on control tissues; however, when provided wounded tissue and LCT, larval mass significantly increased by 47% and 73%, respectively (Figure 2A). Similarly, average (n=11, ±SEM) larval Relative Growth Rates (RGR) demonstrate that control, wounded and LCT diets supported 0.132+0.024, 0.232+0.023, and 0.322+0.017 g g^-1^ d^-1^ of insect growth, respectively. Larvae also consumed nearly 2-fold greater amount of LCT as compared to control tissue; however, this was not significantly different from wounded tissue (Figure 2B). The amount of frass larvae excreted also differed depending on the tissue supplied. When comparing dry frass weights, larvae on LCT diets defecated 2.7-fold and 1.5-fold more than those supplied with control and wounded tissue, respectively (Figure 2C).

ECB larvae provided LCT utilized food more efficiently, as determined by the nutritional indices, efficiency of converted digested food (ECD) and efficiency of ingested food (ECI). Both indices for larvae on LCT were 2-fold greater than respective larvae supplied with control tissues (Table 1). As a potential subtle indicator for defense activation, approximate digestibility (AD) decreased 1.8% in LCT (Table 1). The ECD, ECI, and AD values for ECB given wounded tissue were intermediate between those provided control tissue or LCT (Table 1). When comparing the amount of tissue consumed relative to increased larval mass, larvae supplied with wounded tissue and LCT consumed significantly less than those given control tissue. The consumptive index (CI) was 2.1- and 2.5-fold lower for ECB provided wounded tissue and LCT, respectively (Table 1). As a whole, we interpret the increased larval growth, feeding and above nutritional indices as insect-induced susceptibility in LCT.

**Table 1:** Nutritional indices for ECB larvae on maize stems.

Nutritional Index	Control	Wound	LCT
ECD	0.023 ± .006	0.036 ± .004	0.050 ± .005*
ECI	0.023 ± .005	0.035 ± .004	0.048 ± .005*
AD	0.981 ± .003	0.973 ± .003	0.964 ± .003*
CI	59.286±10.324	28.937 ± 2.907*	23.551 ± 2.985*

Maize stems were either untreated (Control), wounded with a cork borer (Wound) only or additionally infested with an ECB larva for 48 h (LCT). Stem tissues were removed and provided to new larvae for 24 h growth analyses including efficiency of conversion of absorbed food (ECD), efficiency of conversion of ingested food (ECI), approximate digestibility (AD) and consumption index (CI).

* Asterisk denote significant differences from control tissue (*n* = 11 ±SEM; all ANOVAS *P* < 0.01; Tukey test corrections for multiple comparisons, *P* < 0.05).

To assess the nutritional content of theses tissues, quantities of protein, carbohydrates (sucrose, glucose, and fructose) and free fatty acids were measured. At 48 h the total quantity of soluble protein in LCT was 2.6-fold and 1.7-fold greater than control and wounded tissues, respectively (Figure 2D). Interestingly, at 24 h, there was no significant difference in protein quantity between the three treatments (Figure S1B). Given that significant protein increases occur in stems between 24 and 48 h during ECB herbivory, short-term (24 h) feeding studies with excised stems (control and LCT) are well suited for capturing this interaction. When comparing quantities of simple carbohydrates, levels of glucose and fructose did not significantly differ among the three treatments. However, sucrose levels increased nearly 2-fold in both wounded tissues and LCT (Figure 2E and Figure S1C). There were no significant differences observed for the free fatty acids, stearic acid (18:0), oleic acid (18:1), or linolenic acid (18:3) (Figure S1D). However, levels of linoleic acid (18:2) in LCT were 6.9-fold and 2.4-fold greater than control and wounded tissues, respectively (Figure 2F).

### Defense Gene Expression and Proteins are Upregulated in Response to ECB Attack

Although there was a significant total soluble protein increase in LCT, it was unclear if this was due to an increase in transcription and translation of a broad based set of proteins or a highly specific subset. Affymetrix microarray analysis, covering 13,339 genes, revealed a total of 2,028 genes differentially regulated in LCT as compared to untreated control tissue (Table 1, Table S1). Genes for which transcript levels increased 2-fold or more included pathogenesis-related proteins, protease inhibitors, glutathione S-transferases, histones involved in chromatin remodeling, and ribosomes involved in protein synthesis (Table 2, Table S2). Other major gene categories that were differentially regulated were related to auxin and ethylene signaling. Levels of transcripts encoding an auxin binding protein (*Abp20*), a predicted indole-3-acetic acid-amido synthetase (*GH3*) and other proteins that modulate levels of active IAA were highly up-regulated (Table S2). Proteins encoded by rice (*Oryza sativa*) *GH3.8* have demonstrated role in regulating basal immunity to *Xanthomonas* *oryzae* pv *oryzae* in part by reducing free IAA accumulation via aspartic acid conjugation [37]. In general, expression of *AUX/IAA* gene family members were commonly down-regulated while expression of several genes encoding ethylene responsive factors were up-regulated (Table S2).

**Table 2:** Gene categories differentially regulated in ECB-damaged stem tissue.

**Gene Category**	**Up**	**Down**
Total significant	1135	893
Pathogenesis-related	24	0
Protease Inhibitor	13	0
Glutathione S-transferase	19	1
Chromatin remodeling	46	1
Protein synthesis	23	2
Auxin-related	8	14
Ethylene-related	11	3

Selected gene categories significantly regulated in 48 h larval conditioned stem tissues (*n* = 3) compared to untreated stems, determined by a maize genome microarray (13,339 genes).

**Figure 3:**
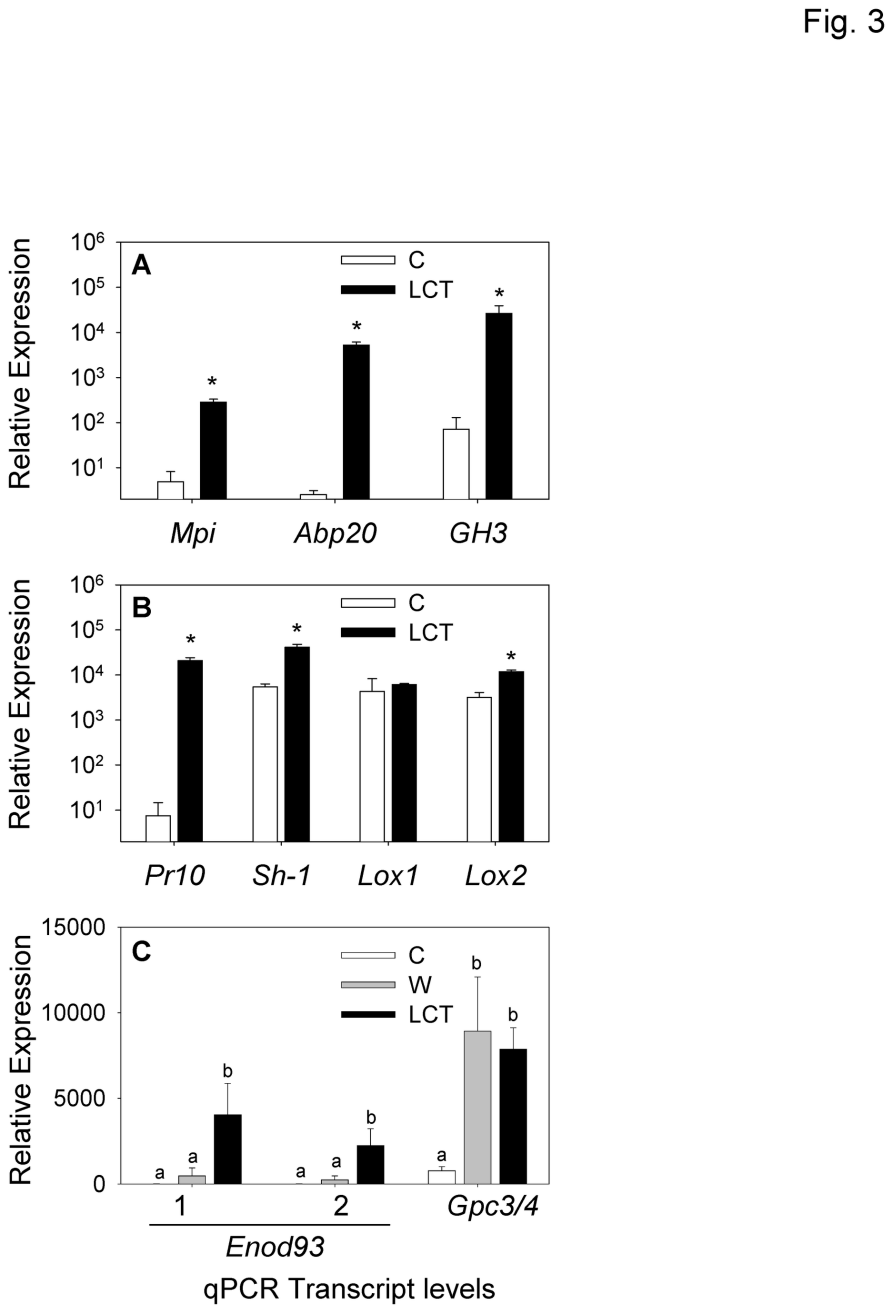
Validation of ECB-induced microarray probe sets with qRT-PCR. **A**, Average (*n* = 3, +SEM) relative gene transcript expression levels for *Mpi*, *Abp20*, and *GH3* which encode the Maize protease inhibitor, Auxin binding protein-20, and a predicted indole-3-acetic acid-amido synthetase. Undamaged control stem tissues (C, white bars) and 48 h larval-conditioned tissue (LCT, black bars). **B**, Similarly, average (*n* = 3, +SEM) relative gene transcript expression levels for *Pr10*, *Sh-1*, *Lox1*, and *Lox2* which encode the proteins Pathogenesis-related 10, Sucrose synthase-1, Lipoxygenase 1 and Lipoxygenase 2. Significant differences indicated by asterisk (Student’s *t*-test, *P* < 0.05). **C**, Average (*n* = 3, +SEM) relative gene transcript expression levels for *Enod93-1*, *Enod93-2*, and *Gpc3/4* encoding two early nodulin 93 proteins and cytosolic glyceraldehyde-3-phosphate dehydrogenase 3/4, respectively for control (white bars), wound (grey bars), and larval-conditioned tissue (LCT, black bars). Different letters (a–b) represent significant differences (all ANOVAs *P* < 0.01; Tukey test corrections for multiple comparisons, *P* < 0.05).

To validate selected microarray results, expression of several strongly up-regulated genes were compared between control tissue and LCT using qRT-PCR. Confirming the microarray results (Tables S1 and S2), the relative expression of transcripts for maize protease inhibitor (Mpi), and the auxin-related genes, *Abp20* and *GH3*, were significantly higher in LCT (Figure 3A). Curiously, an uncharacterized gene annotated as early nodulin 93 (*Enod93*) was among the top 3 most highly induced microarray probe sets (Tables S1 and S2). First characterized in rice (*Oryza sativajaponica*), *OsEnod93-1* exhibits rapid transcriptional activation to both positive and negative changes in nitrate supply rates [38]. Transcript abundance of two related genes, denoted here as *Enod93-1* and *Enod93-2*, was examined by qRT-PCR to address the potential for specificity in ECB elicitation. The expression of both *Enod93* genes was significantly higher in LCT compared to control and wound treatments (Figure 3C).

Isobaric tag for relative and absolute quantification (iTRAQ) was used to identify proteins that were differentially regulated in response to ECB-damage. In a comparison of proteins extracted from untreated control tissue and from LCT, levels of only eight of 169 identified proteins significantly differed (Table 3, Table S3). Proteins for which levels increased at least 1.5-fold included pathogenesis-related protein 10 (PR-10), the lipoxygenases LOX1 and LOX2, and two enzymes involved in carbohydrate metabolism, cytosolic glyceraldehyde-3-phosphate dehydrogenase (GPC3) and sucrose synthase (SH-1). Transcripts encoding PR-10 are highly pathogen inducible and RNAi-silenced maize lines suppressed in PR-10 accumulation are significantly more susceptible to mycotoxigenic fungi [39,40]. LOX1 is wound inducible and has the potential to contribute to JA biosynthesis given dual positional specificity in production of both 9- and 13-hydroperoxides of linolenic acid [41]. LOX2 specificity is unknown; however transcript accumulation is dependent on JA biosynthesis [42]. In contrast to these proteins, levels of α- and β-tubulin were decreased at least 1.5-fold.

**Table 3:** Proteins differentially regulated in response to 48 h of ECB feeding damage.

**Protein Name**	**Accession**	**Predicted kDa/ pI**	**Ratio**	***P* value**
*Up-regulated proteins*				
Pathogeneis-related protein 10	AAY29574	16.9/5.4	2.645	0.0015
Lipoxygenase 2	ABC59686	98.3/6.2	1.561	0.0003
Lipoxygenase 1	ACG43480	98.1/6.2	1.952	0.0003
D-3-phosphoglycerate dehydrogenase	XP_002447228	64.4/6.5	1.761	0.0008
Glyceraldehyde-3-phosphate dehydrogenase	Q43247	63.4/7.0	2.211	0.0036
Sucrose synthase	CAA26229	91.7/5.9	2.159	0.0008
*Down-regulated proteins*				
Alpha-tubulin	CAY56347	53.2/4.9	0.588	0.0005
Beta-tubulin	CAY56281	52.6/4.8	0.656	0.0000

Of the 169 proteins identified using iTRAQ, eight displayed significant differential regulation in 48 h larval conditioned stem tissues compared to respective untreated maize stems (*n* = 4).

To determine whether protein increases were reflected at the transcriptional level, expression of the genes encoding *Pr10*, *Lox1*, *Lox2*, *Sh-1, Gpc3/4* were analyzed by qRT-PCR. *Pr10* transcript levels were greatly increased in 48 h LCT (Figure 3B), and expression of both *Sh-1* and *Lox2* was also significantly greater (Figure 3B). Examination of the microarray data revealed that with the exception of *Sh-1*, probe sets corresponding to each gene were significantly up-regulated (Table S1). In maize, *Gpc3* and *Gpc4* transcript levels are co-regulated by anoxia stress and encode proteins that are 99.4% identical at the amino acid level [43]. To determine whether induced expression of *Gpc3/4* was specific to ECB-damaged tissue, a primer set was designed to recognize both of these related transcripts in control, wound, and LCT treatments. Unlike the *Enod93* genes, the expression of *Gpc3/4* was also significantly up-regulated by wounding alone and thus not specific to LCT (Figure 3C).

### ECB Damage and Wounding Changes the Morphology of Nuclei in Maize Stem Tissue

**Figure 4:**
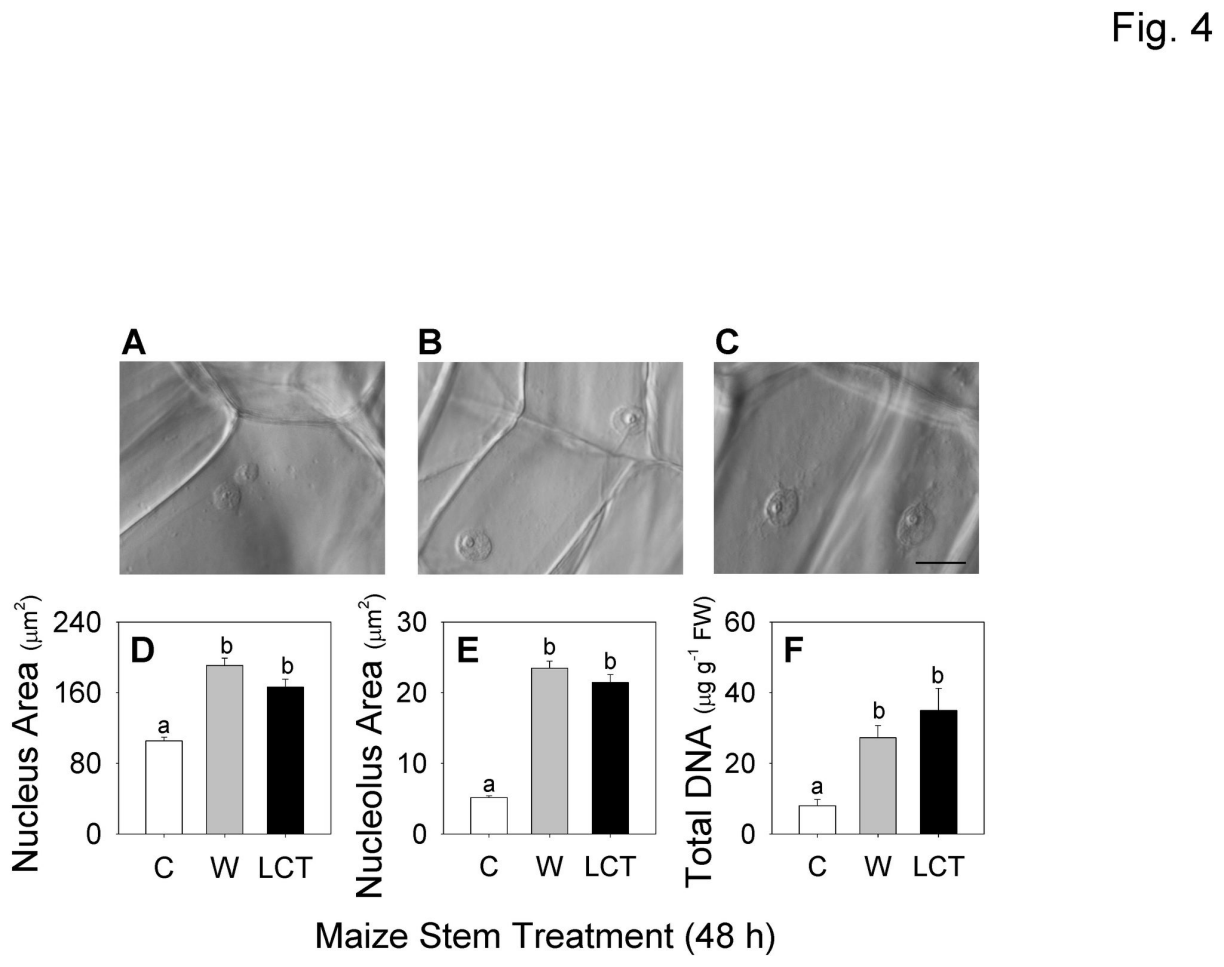
Consistent with maize endoreduplication, both wounding and ECB stem herbivory treatments promote significant increases both nuclear and nucleolar size as well as total DNA levels. Light microscopy pictures of nuclei and nucleoli from representative **A**, control (C), **B**, wounded (W) and **C**, larval-conditioned tissues (LCT). Scale bar = 20 µm. Average areas (*n* = 40, ±SEM) of **D**, nuclei and **E**, nucleoli and **F**, concentration of total DNA (*n* = 3, +SEM). Different letters (a–b) represent significant differences (all ANOVAs *P* < 0.01; Tukey test corrections for multiple comparisons, *P* < 0.05).

Endoreduplication, a process in which cells undergo DNA replication without concomitant mitosis, is one mechanism that could result in elevated tissue levels of total protein [44–46]. To ascertain whether ECB-induced increases in protein content were associated with endoreduplication, the area of nuclei in 2D digital micrographs was compared between control, wound, and LCT treatments (Figure 4A–C). Nuclei were approximately 1.6-fold larger in both wounded tissue and LCT as compared to controls (Figure 4D). Moreover, the size of the nucleolus exhibited significant 4-fold increases in both wounded tissue and LCT (Figure 4E). There were no significant differences in either nucleus or nucleolus size between LCT and wounded tissues. Absolute quantification of DNA within nuclei was not possible due to the lack of a reliable internal standard and interference from non-specific staining of cell walls by fluorescent DNA stains. However, consistent with predictions for endoreduplication, total extractable DNA content was also approximately 4-fold greater after wound and LCT treatments compared to untreated control tissues (Figure 4F).

### ECB Excretions and Secretions Contain Elevated Levels of Indole-3-Acetic Acid

**Figure 5:**
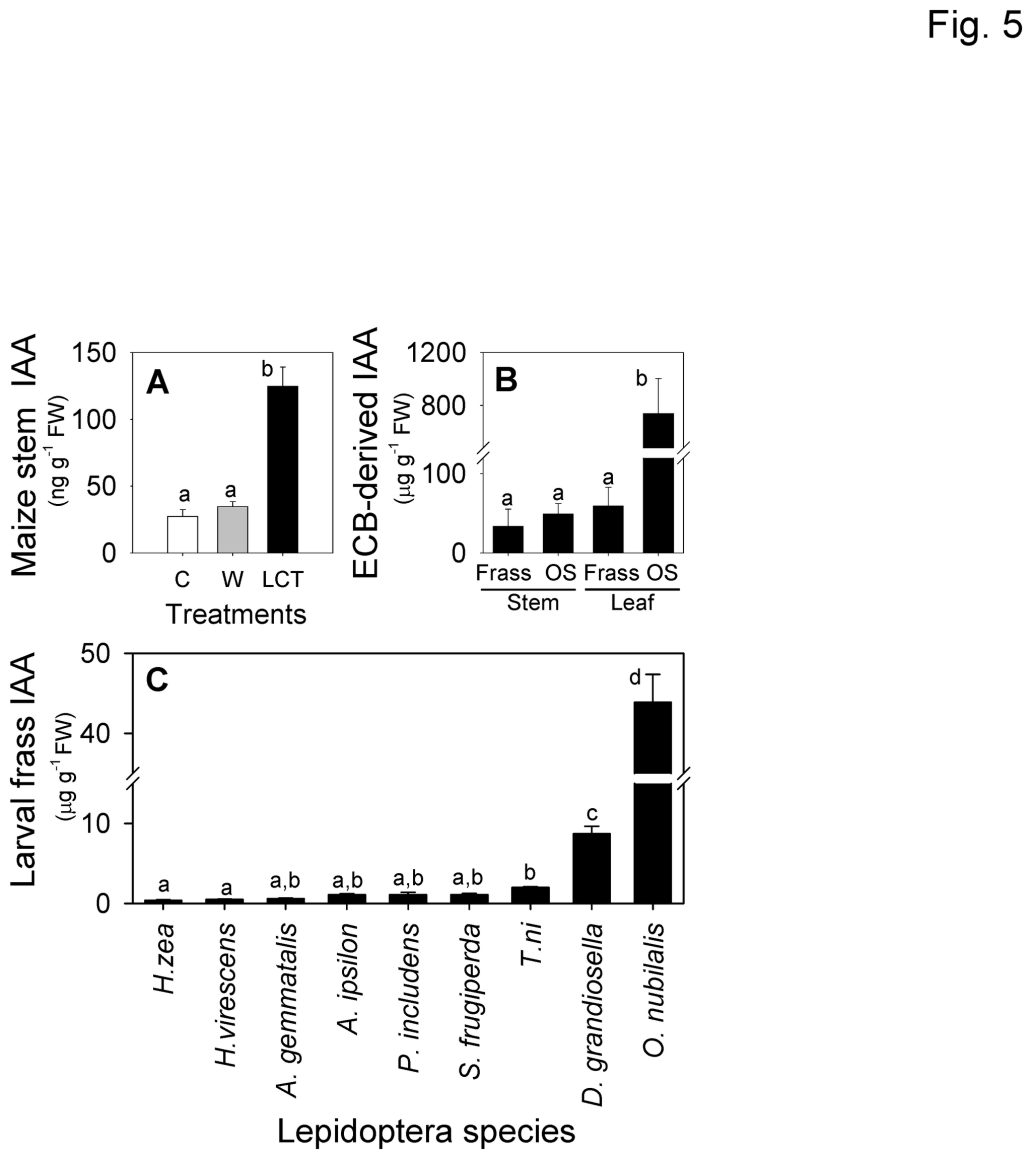
ECB oral secretions and frass contain unusually high levels of IAA. **A**, Average (*n* = 3, +SEM) concentration of IAA in maize stem tissue collected from control (C), wounded (W), and ECB larvae-conditioned tissues (LCT) after 48 h. **B**, Average (*n* = 4, +SEM) IAA levels in ECB OS or recently collected (1 h) frass following larval ingestion of maize leaf or stem tissue for 24 h. **C**, Average (*n* = 3, ±SEM) IAA in frass of numerous Lepidopteran pest species ( *Helicoverpa* *zeae* , *Heliothis* *virescens* , *Anticarsia* *gemmatalis* , *Agrotis* *ipsilon* , *Pseudoplusia* *includens* , *Spodoptera* *frugiperda* , *Trichoplusia ni*, *Diatraea* *grandiosella* and *Ostrinia* *nubilalis* : ECB) reared on artificial diet. Different letters (a–d) represent significant differences (all ANOVAs *P* < 0.01; Tukey test corrections for multiple comparisons, *P* < 0.05).

In terms of protein levels, ECB-damage has a more pronounced effect on maize stems than wounding alone. In a previous analysis of rapid phytohormone changes in stem tissue, ECB attack resulted in significant 3-fold increases IAA concentrations within 3 h [5]. This response was specific and did not occur after wounding alone. Consistent with this rapid dynamic, LCT exhibits 3.5-fold greater IAA levels than wounding alone, even after 48 h (Figure 5A). Surprisingly, a screen for candidate small molecule effectors in ECB OS revealed exceedingly high levels of free IAA. ECB OS from larvae supplied with leaf and stem tissue displayed IAA concentrations of 800 and 50 µg ml^-1^, respectively (Figure 5B). Levels of IAA in freshly collected frass samples from larvae provided leaf and stem diets were also greatly elevated (Figure 5B). In air-dried frass samples, levels of IAA were not statistically different than those in leaf OS. The average (*n* = 3) quantity of IAA in dry frass samples originating from leaf and stem diets is 951 ± 382 µg g^-1^ and 872 ± 582 µg g^-1^, respectively.

To determine the prevalence of IAA in the predominant excretions of other Lepidopteran species, freshly generated frass (1 h) was collected from *H* *. zeae* , *Heliothis* *virescens* , *Anticarsia* *gemmatalis* , *Agrotis* *ipsilon* , *Pseudoplusia* *includens* , *S.* frugiperda, *Trichoplusia ni*, and *Diatraea* *grandiosella* larvae supplied with an artificial diet used for routine rearing. ECB frass contained significantly greater levels of IAA than all other species examined; however, elevated IAA was not specific to ECB frass (Figure 5C). The only other stem borer examined, *D* *. grandiosella* (southwestern corn borer), had the second highest frass IAA concentration with significantly greater levels than the other seven species. Given that ECB frass and OS IAA levels range from 40–800 µg g^-1^ FW and that stem tissue concentrations of this phytohormone increase by 100 ng g^-1^ FW during ECB attack (Figure 5A–C), the 400-8,000 fold higher IAA levels in ECB excretions/secretions provide a parsimonious source and explanation for the increased levels. However, our current analyses are unable to distinguish between plant, insect, or microorganism derived IAA.

### Sustained Exposure to a Stable Synthetic Auxin Elevates Stem Protein Levels

**Figure 6:**
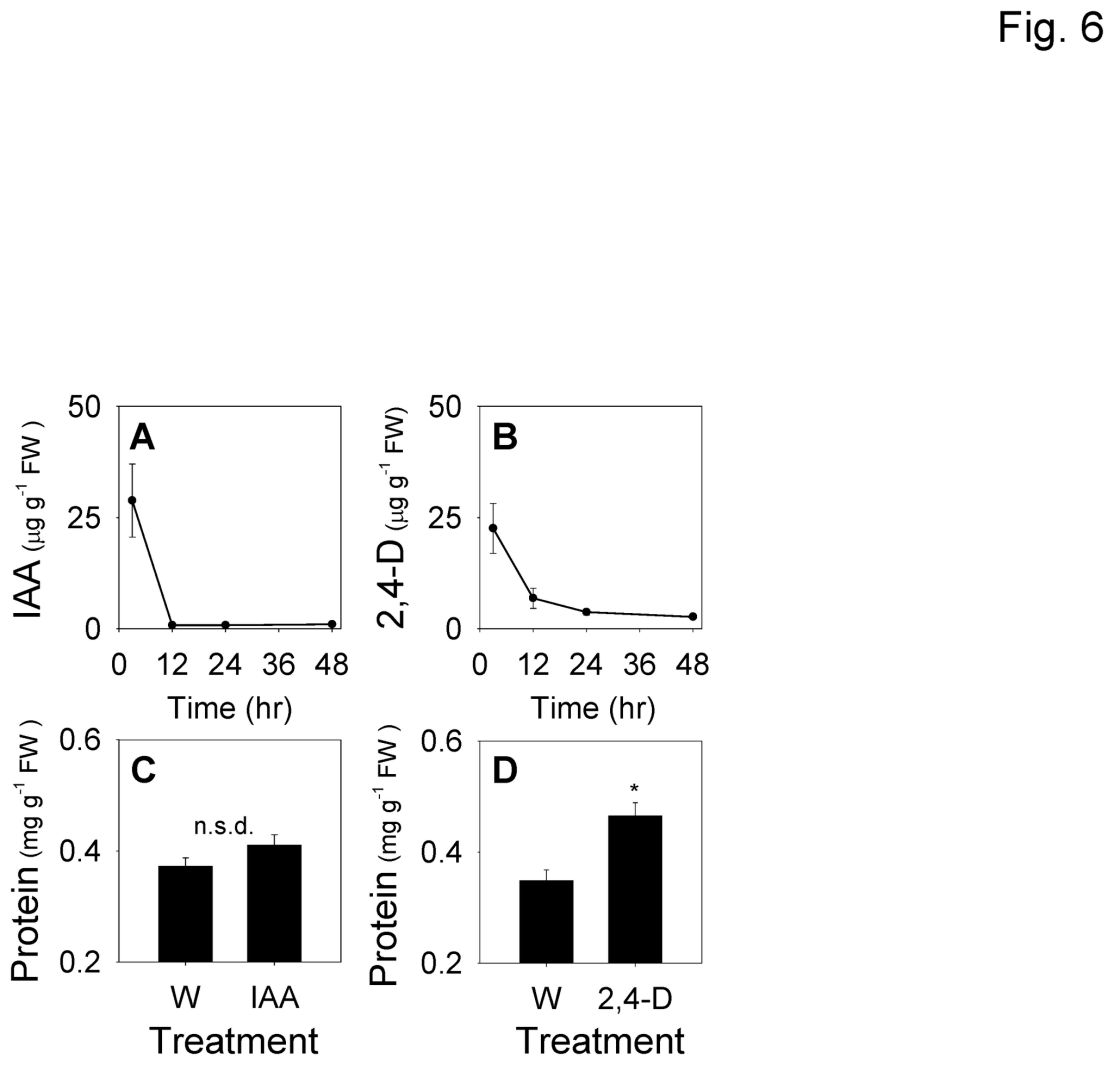
Prolonged exposure to a synthetic stable auxin increases total protein levels in maize stem tissue. Average (*n* = 4, ±SEM) auxin levels of **A**, indole-3-acetic acid (IAA) and **B**, 2,4-dichlorophenoxyacetic acid (2,4-D) following a 50 µg application to wounded (W) stems and subsequent harvests at 3, 12, 24, and 48 h after treatment. Average (*n* = 6, +SEM) total soluble protein extracted at 48 h from maize stem tissue treated with 50 µg **C**, IAA or **D**, 2,4-D. Significant differences are indicated by asterisk (Student’s *t*-test, *P* < 0.05; n.s.d = no statistical difference).

Through contamination with frass and OS, growing ECB larvae are likely to supply a continuous source of IAA to the feeding tunnel. In an effort to replicate this system, IAA and the synthetic auxin analog, 2,4-dichlorophenoxyacetic acid (2,4-D), were applied to wound sites in maize stems and the metabolism of these compounds were measured over a 48 h time period. Compared to synthetic IAA, exogenous applications of 2,4-D to plant tissues commonly display lower rates of inactivation via conjugation and likewise greater biological activity [47]. In the current study, free IAA applied to the stem was completely metabolized within 12 h (Figure 6A). In contrast, although levels of 2,4-D substantially dropped after 12 h, concentrations of this auxin remained significantly elevated even 48 h after application (Figure 6B). When IAA was applied to wounded maize stem tissue, total protein content did not differ from wounded tissue after 48 h (Figure 6C). In contrast, application of 2,4-D to wounded stems resulted in a significant 25% increase in total soluble protein (Figure 6D). This auxin induced increase is consistent with the elevated stem protein levels present in ECB challenged stems (Figure 2D).

## Discussion

Historically ECB have been among the most devastating insect pests affecting commercial maize production [6]. Upon hatching, larvae feed upon the whorl tissue and eventually bore into the stalk where they cause the majority of damage, lodging and economic loss. In leaves of young maize plants, the benzoxazinoid DIMBOA-Glc is closely associated with resistance to ECB herbivory while in mature stems, resistance appears to be associated with cell wall composition [35]. Specifically, high stem concentrations of xylose and diferulates have been negatively correlated to ECB tunnel length [48]. In response to ECB damage, lignin content increases in maize stems, as do levels of the benzoxazinoid HDMBOA-glc and kauralexins [5,49]. HDMBOA can negatively impact the growth and development of insects including the rose-grain aphid ( *Metopolophiumdirhodum* ) and *D* *. grandiosella* [50,51]. Similarly, anti-feedant activity has been demonstrated for HDMBOA against fall armyworms and kauralexins against ECB [8,36]. Despite numerous induced-defense responses at 24 h, short-term growth of ECB larvae is not significantly altered [5]. To assess efficacy of longer-term plant responses, ECB were supplied stem tissues previously subjected to 48 h of ECB herbivory. In the current study, resulting stem tissues contained greater levels of total benzoxazinoids and kauralexins than those previously observed at 24 h [5]. We hypothesized that elevated defenses associated with long-term attack could decrease tissue quality and likewise ECB growth. Surprisingly, larvae gained significantly more weight following consumption of LCT compared to control or wounded tissues. Microarray analyses confirmed that numerous defense-related genes were up-regulated in LCT compared to control tissue and shared significant overlap with those reported for the Mediterranean corn borer ( *Sesamia* *nonagrioides* ) [52]. Although the expression of many defense-related genes were induced in response to ECB, a correspondingly broad increase proteins was not observed by iTRAQ analysis. This is likely due to limitations of the current analysis which resulted in positive identification of only 169 proteins which represents a small fraction of the anticipated diversity. Of those identified, levels of three established pathogen- and wound-inducible proteins significantly increased in LCT, including PR-10 and two lipoxygenases, LOX1 and LOX2 [40–42]. A lack of strongly induced insect-related defense proteins could render maize stalks susceptible to ECB herbivory [12].

Significant maize resistance against ECB requires high levels of structural or biochemical defenses. Maize lines rich in benzoxazinoids, ranging from 200–700 µg g^-1^ FW, generally display measurable ECB resistance in the whorl tissue while those with less than 100 µg g^-1^ FW are typically susceptible [53]. Consistent with this result, ECB larval growth was unaltered in longer-term artificial diets containing 50 µg g^-1^ FW DIMBOA [54]. In the current study, ECB stem attack significantly increased the levels of HDMBOA-glc; however, the total pool of benzoxazinoids remains comparatively modest (≤ 5 µg g^-1^ FW) and likely insufficient to promote resistance. Related pest species, such as the Asian corn borer ( *Ostrinia* *furnacalis* ), are able to tolerate significant levels of xenobiotics and detoxify even methanol, formaldehyde and formic acid at levels exceeding 1% of the diet [55]. In contrast to induced plant resistance, previously attacked maize stems became more nutritious and sustained improved ECB growth. Larvae supplied with LCT displayed higher efficiencies for conversion of absorbed food (ECD) and ingested food (ECI) indicating that the insects are obtaining more essential nutrients than those provided with control and wounded tissues [56]. ECI values increase in relation to the nitrogen content of the plant tissue and total nitrogen content increases in maize stem tissue subjected to ECB-herbivory [49,57]. Curiously, two *Enod93* genes were specifically up-regulated in LCT. While the precise role of these genes is not currently known, the constitutive expression of *Enod93* in transgenic rice resulted in significantly higher levels of total amino acids in moderate to low nitrate environments [38]. Most nitrogen in the plant is incorporated into proteins, thus ECB-induced *Enod93* expression might lead to higher amino acid levels that would permit increased protein synthesis. Increased levels of free amino acids are also observed following root herbivory by western corn rootworm ( *Diabroticavirgifera* ) larvae and likewise increased plant susceptibility to conspecific larvae [4]. Modest increases in total protein have also been reported in rice damaged by the yellow stem borer ( *Scirpophaga* *incertulas* ) [58].

A potential role of ECB-excreted IAA is the suppression of wound induced defense responses. In tobacco, exogenous IAA can effectively inhibit both wound-induced JA accumulation and subsequent nicotine synthesis associated with jasmonate signaling [30]. In maize, the inducible accumulation of both HDMBOA-glc and kauralexins has been shown to be positively regulated by the synergistic activities of JA and ET [5,8]. In previous work and the current study, both kauralexins and HDMBOA-glc are significantly higher in LCT than respective mechanical damage controls [5]. However, ECB-induced JA and ET levels remain significantly elevated compared to wounding alone even in the presence of elevated IAA [5]. In contrast to tobacco, exogenous application of the synthetic auxin 2,4-D in rice elicited increases in JA, ET, defense gene expression, direct herbivore resistance and parasitoid attraction [59]. Select chlorinated herbicides such as 2,4-D may function through the activation of auxin signaling and also by altering cellular redox levels that subsequently trigger detoxification responses [60–62]. Taken as a whole, our results in maize do not support the hypothesis that ECB associated IAA significantly inhibits the activation of biochemical responses. The significant transcript accumulation for both pathogen and insect-related defense is anticipated given the combined biotic challenges that occur during stem boring [7]. Importantly, LCT contains increased levels of total soluble protein; however, iTRAQ analysis revealed only six proteins with statistically significant accumulation. These candidates alone are unlikely to account for the 2.6-fold increases in LCT total protein; therefore, it is likely that a diverse portion of the proteome increased in response to insect attack. Consistent with this hypothesis, expression of many genes associated with protein synthesis were significantly up-regulated by ECB herbivory as analyzed by microarray, with twenty-three genes encoding ribosomal proteins alone. Significant enlargement of the nucleus and nucleolus is also supportive of large scale increases in protein synthesis. As the site of ribosomal RNA synthesis and ribosome assembly, an increase in nucleolus size and ribosomal proteins facilitates the additional translational demands of the cell [63].

With increased expression of genes encoding enzymes for a given metabolic pathway, metabolic pathway flux also commonly expands [64]. Likewise, the up-regulation of sucrose synthase at both the RNA and protein level correlated to increased quantities of sucrose in LCT as compared to control tissue. Levels of free linoleic acid also increased in LCT which parallels observations for gall-inducing insects such as the Hessian fly ( *Mayetiola* *destructor* ) and the caterpillar *Gnorimoschema* *gallaesolidaginis* [65,66]. The lipoxygenases, LOX1 and LOX2, were up-regulated in response to ECB herbivory and likely act on induced pools of free linoleic acid as substrates for oxylipins [67]. Interestingly, increases in both sucrose and linoleic acid in LCT could also function as ECB phagostimulants. A combination of simple sugars (sucrose, glucose, and fructose) and linoleic acid significantly increased western corn rootworm herbivory levels more than either sugars or lipids alone [68]. Although insects generally exhibit compensatory feeding on nutrient-poor tissues, ECB consumed higher amounts of LCT despite the significantly increased nutritional content. This indicates that the increased rate of herbivory was not compensatory [57]. Overall, an increase in both primary and secondary metabolism is consistent with large-scale metabolic changes observed following herbivore attack [69].

Endoreduplication occurs when ploidy of the cell increases due to replication of the genome without concomitant mitosis, which can result in broadly increased translation of the proteome and thus increased metabolic flux [44–46]. Developmentally-regulated endoreduplication occurs in a wide range of plant tissues, but is also induced by bacterial symbionts, fungal pathogens, and nematode damage [45,46]. Analysis of transcriptome data from plant tissue that has undergone endoreduplication reveals significant accumulation of transcripts associated with chromatin remodeling and protein synthesis [46]. A similar set of genes with up-regulated expression was observed in LCT (Table 2). Although we were unable to directly compare the ploidy between control and treated tissues, significant increases in nuclear area, nucleolar area, total DNA content and total protein are consistent with wound and ECB-induced endoreduplication in stems. It has been suggested that plants undergo endoreduplication to meet the increased metabolic demands imposed by organisms interacting with the plant [46]. This process may be essential to ECB-induced responses in maize stem tissue.

ECB-induced increases in total DNA, nuclear and nucleolar size appear to be largely regulated by the plant response to wounding. However, responses such as protein increases are significantly more pronounced in LCT suggesting a possible contribution of arthropod-associated plant effectors. One hypothetical effector candidate is the phytohormone IAA, which is present at high concentrations in ECB frass and OS. With the exception of *D* *. grandiosella* , an ECB-related Pyraloidea superfamily member, ECB frass contained >20-fold higher levels of IAA compared to 7 other Lepidoptera pest species consuming a standard artificial diet for rearing (Figure 5C). Through deposition of frass during stem herbivory, ECB are likely to provide IAA to the surrounding feeding tunnel and plant tissues. To mimic the effects of sustained IAA application in maize stalks, we utilized the synthetic analog, 2,4-D. Although IAA has slightly higher affinity for the F-box protein receptor, Transport Inhibitor Response 1 (TIR1), 2,4-D also interacts with TIR1 to promote downstream signaling [70]. Unlike 2,4-D, IAA applied to maize stems was completely metabolized within 12 h. Herbivore-deposited IAA is also likely to be metabolized quickly by plant tissue and deactivated via conjugation by GH3 enzymes encoded by transcripts observed to be highly expressed in LCT [37,71]. Due to rapid deactivation, a single application of IAA to maize stem tissue is not sufficient to impact total soluble protein content (Figure 6A and C). It seems that sustained auxin exposure might be required to further increase wound-induced protein levels. Correspondingly, treatment of plants with a more slowly metabolized analog, such as 2,4-D, better mimicked sustained auxin exposure and resulted in significantly increased protein content (Figure 6B and D). These results are consistent with a previous study by Oka and Pimentel, in which maize plants treated with 2,4-D displayed increased protein levels and greater pupation weights for ECB larvae reared on these plants [72]. In field trials, plants exposed to 2,4-D were also infested with ECB at significantly higher rates than unexposed control plants. Similarly, rice plants treated with 2,4-D displayed increased susceptibility to the striped stem borer ( *Chilo* *suppressalis* ) [73]. While potentially informative, the xenobiotic detoxification processes additionally activated by 2,4-D do not directly relate to IAA signaling and collectively complicate interpretation of these results in the context of naturally occurring auxins [62]. Mindful of these complications, as a future testable hypothesis we speculate that the artificially-induced susceptibility mediated by synthetic auxin spraying may be relevant to a naturally occurring micro-scale mechanism that contributes to ECB success against maize.

In contrast to these results, the root-based application of 2,4-D to rice plants increased JA, ET, and transcript levels of numerous defense markers which resulted in a decreased the growth 2^nd^ instar stem borer larvae over a 6 day period [59]. In an attempt to reconcile the finding of this recent rice study and those performed in maize we consider the following experimental differences. In Xin et al., the root systems of hydroponically grown rice were treated with 2 mg L^-1^ 2,4-D for multiple days and *C* *. suppressalis* growth on leaves was monitored [59]. In the present study, surprisingly high levels of IAA were found in maize internode tissues where ECB larvae were feeding. This resulted in the local examination of exogenous IAA and 2,4-D activity at this site with respect to total protein increases previously witnessed during insect feeding. A single application of IAA to stem tissues was rapidly metabolized by the plant and did not promote protein increases above mechanical damage alone (Figure 6A,C). Collectively, differences in plant genera examined, chemicals used, tissues treated, insects assayed, and time frame of the experiments undoubtedly influenced the observed results. Our primary interest was to first characterize the plant responses that occur following actual ECB stem attack. Having first observed improved ECB growth on LCT, which equates to an induced susceptibility, we then found that ECB OS and frass contain significant levels of IAA.

High levels of IAA have not been previously reported in the frass or OS of any Lepidopteran crop pest; however, plant parasitic nematodes are known to manipulate auxin signaling. To establish feeding sites, nematodes secrete an effector that modifies auxin transport resulting in highly localized IAA accumulation [74]. The precise source of IAA in ECB secretions is not known at this time. Auxin biosynthesis is common in microorganisms, and while not identified in insect secretions, bacteria isolated from the gut of the diamondback moth ( *Plutellaxylostella* ), were capable of producing IAA [75]. Recently, gall-inducing sawfly in the genus *Pontania* have been demonstrated to synthesize 20 ng IAA from 1 µg of isotopically labeled tryptophan; however, the source of this metabolic conversion remains unknown [76]. Given the broad host range, economic relevance, and high levels of auxin produced, we propose ECB larvae as an excellent model system to study the biosynthetic origin of IAA in Lepidoptera. Comparative experiments with other Lepidopteran pests that excrete trace amounts of IAA may also aid the understanding of this interaction.

ECB have long been recognized as dramatically damaging pests of maize with leaf feeding by even early instars significantly altering plant growth [6,7,77]. Over 50 years ago, Chiang and Holdway conducted a 4 year field study examining maize internode elongation during controlled infestations with ECB larvae [77]. The authors conclude that “The reduction in the length of internodes began before the borer entered the stalk and at a time when only the leaf blades were injured. Therefore the effect of the borer infestation on the elongation of the internodes must have been brought about by processes other than physical destruction or obstruction of the vascular bundles in the stalk. This fact suggests that the borer feeding on leaf blades could have been responsible for or could have initiated some chemical change which reduces the normal processes of growth, particularly elongation, of the internodes as well as of the leaf blade itself. The chemical changes may involve a phytotoxic secretion produced by the borers or by micro-organisms which may be associated with the borers” [77]. In the final sentence Chiang and Holdway state “The present study has uncovered enough facts to indicate the need for further studies along the lines of plant anatomy, plant physiology, and plant chemistry in association with studies on insect biology”. Advances in analytical biochemistry and molecular biology now make these studies possible.

In Arabidopsis, herbivores such as *P* *. brassicae* and *S* *. littoralis* contain unidentified OS effectors that suppress plant defense responses [3]. While ECB are unable to attenuate stem defense responses in a similar manner, they are able to improve subsequent growth and functionally induce susceptibility. Nutritional indices demonstrate that ECB-stimulated increases in total protein are sufficient to override the negative effects of plant chemical defenses. During foliar ECB herbivory, IAA levels in the OS can be remarkably high, approaching a concentration of 0.1%, and ECB-induced increases in stem nutritional content can be mimicked in part by application of auxins, such as 2,4-D. As an added challenge to understanding signaling and plant responses associated with continuous mechanical damage [78], thus far experimentally mimicking multiple days of sustained cryptic tunneling by ECB larvae has proven difficult. Clearly mechanical damage initiates many of the responses associated with ECB attack; however, the elevated levels of IAA detected in larval OS, frass and stem tissues results in a future testable hypothesis that insect associated auxin may promote further increases in tissue protein accumulation witnessed during herbivory. IAA is deployed by numerous phytopathogenic micro-organisms; however, it has not been previously hypothesized to act as a potential effector in agronomically important herbivores [79]. Our work raises the hypothesis that IAA may exist as a ECB effector, deployed by larvae to condition maize tissues for optimal insect growth. Further experiments aimed at understanding the regulation of ECB-associated IAA production and ultimately the generation of larvae lacking IAA will provide essential tools for this area of research.

## Materials and Methods

### Plant and Insect Materials

Seeds of *Zea mays* (var. Golden Queen) were germinated and maintained as previously described [5]. Briefly, plants were grown in a greenhouse (12 h photoperiod) and temperature was maintained at 24 °C: 28 °C (night: day). Plants were watered daily and fertilized weekly with 1.25 mg L^-1^ Peters 20-20-20 water soluble fertilizer (Scotts). Plants used for studies were approximately 30 days old and had 11-13 leaves. *Ostrinia* *nubilalis* , *Helicoverpa* *zeae* , *Heliothis* *virescens* *,* *Anticarsia* *gemmatalis* , *Agrotis* *ipsilon* *,* *Pseudoplusia* *includens* *,* *Spodoptera* *frugiperda* , *Trichoplusia ni* and *Diatraea* *grandiosella* larvae were supplied by Benzon Research (Carlisle, PA). Third to 5^th^ instar ECB larvae are established pests that tunnel into maize stalks [80]. Prior to infesting plants with ECB, newly molted 5^th^ instar larvae were provided maize whorl tissue for 24 h to simulate the transition from leaf to stem feeding.

### Experimental Treatments

For the microarray and iTRAQ studies, ECB were introduced into leaf sheaths and allowed to bore into the stem. Samples were only collected from plants where ECB had tunneled into developmentally similar stem internodes for 48 h, creating larval conditioned tissues (LCT).

To reduce the variation associated with small sample sizes, each replicate was derived from 3 separate individual plants within each treatment group. For example, 9 individual plants were required generate 3 analytical samples (n=3). Corresponding tissue samples were also collected from plants that were not previously damaged. To better control routine establishment of ECB tunneling, a #1 cork borer was used to create a hole in the stem internode tissue and larvae were encouraged to enter this site using a modified 200 µL pipette tip [5]. At indicated time points, approximately 2 cm stem sections were collected around the initial site of cork borer damage (wound treatment) and established ECB feeding tunnels. Similar untreated control tissues were also collected from untreated plants. Epidermal tissue was uniformly avoided by larvae that had established feeding tunnels; thus, epidermal tissue was excluded from all internode tissue analyses. For application of indole-3-acetic acid (IAA) and 2,4-dichlorophenxyacetic acid (2,4-D), a hole was made with a #1 cork borer in the middle of the second and third nodes from the base of the stem and a 50 µl solution containing 1µg µl^-1^ IAA or 2,4-D prepared in 5% (v/v) methanol/ water was applied to the wound site. Wound controls were treated with an identical solution lacking auxins. The wound site was sealed with parafilm to reduce drying and samples were collected at 48 h.

### Gene Expression Analysis

RNA from stem tissue was extracted using a modified TRIzol® reagent (Invitrogen, Carlsbad, CA) protocol described previously [5]. Two µg of RNA were reverse transcribed into cDNA using RETROscript® First Strand Synthesis Kit (Applied Biosystems, Carlsbad, CA). cDNA was diluted 10-fold prior to quantitative real-time PCR (qRT-PCR) which was performed and analyzed as previously described [5]. Relative expression levels were determined for three independent biological replicates each of which consisted of a pool from three separate plants and all the reactions were run in triplicate. Threshold cycle (Ct) values for *early nodulin 93-1* (*Enod93-1*), *early nodulin 93-2* (*Enod93-2*), and *indole-3-acetic acid-amido synthetase* (*GH3*) were normalized to *histone deactelyase* (HD). The values for *Pathogenesis-related 10* (*Pr10*)*, Sucrose synthase-1*(*Sh-1*)*, Lipoxygenase 1* (*Lox1*) *Lipoxygenase 2* (*Lox2*)*,* Maize protease inhibitor (Mpi) and Auxin binding protein *20* (*Abp20*) were normalized to the housekeeping gene encoding *eukaryotic translation initiation* factor *4B* (*ETIF*). The Ct values for cytosolic *glyceraldehyde-3-phosphate dehydrogenase-3/4* (*Gpc3/4*) were normalized to *Ribosomal protein L17* (*RpL17*). The levels of each gene transcript were calculated relative to its corresponding untreated control. Fold-changes of RNA transcripts were calculated by the 2^-ΔΔCt^ method [81]. The specificity of real-time PCR products were verified with dissociation curves. The gene-specific oligonucleotides are listed in Table S4.

### Microarray analysis using Affymetrix GeneChip Maize Genome Arrays

RNA was extracted from 48 h untreated control and ECB-damaged internode tissues as described above. Three biological replicates, each replicate consisting of 3 individual plants pooled, were analyzed for each treatment and samples were DNase treated prior to Affymetrix GeneChip maize genome array (13,339 genes) analyses performed by the University of Florida Interdisciplinary Center for Biotechnology Research Gene Expression Core. Samples were prepared and data was analyzed as previously described [40]. The microarray analysis (GSE46475) has been submitted to the NCBI-Gene Expression Omnibus (http://www.ncbi.nlm.nih.gov/geo/query/acc.cgi?acc=GSE46475) as a permanent repository.

### Biochemical Analyses

Benzoxazinoids were quantified using reverse phase HPLC as previously described [82]. Concentrations of 18-carbon free fatty acids, A- and B-series kauralexins, IAA and 2,4-D were measured using the vapor phase extraction coupled with gas chromatography / chemical-ionization mass spectrometry (GC/CI-MS) described by Schmelz et al. [8,83]. For the analysis of ECB OS, 4 individual 5^th^ instars previously feeding on plant tissue for 24 h were gently pinched with tweezers causing a regurgitation of 1-3 µL of OS per larvae which was collected into capillary tubes. Each sample was derived from a separately collected OS pool of approximately 5-10 µL each. From each separate pool, a 2 µL OS aliquot was added to 100 µls of 9:1 MeCl_2_: MeOH immediately prior to derivatizing the sample with trimethylsilyldiazomethane and subsequent GC/CI-MS analysis [8,83]. Frass from Lepidoptera larval reared on leaf, stem and standardized artificial diet (Benzon Research) were collected from many individuals within 1h of excretion to create individual 50 mg samples which were then processed identically to a plant samples. Larvae were held on these diets for at least 24 h prior to frass collection. Glucose, fructose and sucrose concentrations were quantified as previously described [84,85]. Total soluble protein was extracted from 0.5 g stem internode tissue in 1 ml protein extraction buffer (10 mM Na_2_HPO_4_, 15 mM NaH_2_PO_4_, 100 mM KCl, and 2 mM EDTA). Samples were centrifuged for 15 min at 10,000 x g at 4^°^C and quantity of protein in the supernatant was determined using the Quick Start™ Bradford Dye Reagent (BioRad, Hercules, CA) [86]. Proteins were extracted from a total of six biological replicates for control, wound, and LCT treatments.

### iTRAQ Protein Separation and Data Analysis

Samples for iTRAQ were processed and analyzed as previously described with the following modifications [87]. Peptides were labeled using the iTRAQ Reagents Multiplex kit (applied Biosystems, Foster City, CA). Peptide samples from four control biological replicates were labeled with iTRAQ tags 113, 114, 115, 116 and peptide samples extracted from LCT were labeled with iTRAQ tags 117, 118,119 and 121 (Fig. S2). Equal quantities of protein were used for each treatment and the protein fold changes were relative to a control biological replicate labeled with the 113 isobaric reagent. The MS/MS Data was processed by a thorough search considering biological modifications against NCBI green plants FASTA database (2,841,664 entries, downloaded on September 9, 2009) using the Paragon algorithm of ProteinPilot v 4.0 software suite (Applied Biosystems, USA) [88]. As a conservative estimate of differential expression, a protein had to be quantified with at least three spectra (allowing generation of a *P*-value), a *P* <0.05, and a ratio fold change of at least 1.5 in more than two independent experiments.

### No Choice Feeding Assay with ECB

As described above, stem sections (2 cm) were collected from control, wound, and LCT treatments after 48 h. Tissue was weighed and placed into sterile 12-well tissue culture plates (Fisher Scientific, Vernon Hills, IL). For each stem section, a newly molted 5^th^ instar ECB previously maintained on artificial diet was weighed and placed on the stem tissue. After 24 h, the stem tissue, ECB larvae, and frass were weighed to determine the amount of tissue consumed and the percent weight gain for each ECB. A total of 11 ECB were used for each treatment. The nutritional indices were measured as described in Scott et al. [56]. The indices calculated included the consumption index (CI = stem mass ingested / (larval mass gain * number of days)), approximate digestibility (AD = (stem mass ingested – frass)/ stem mass ingested), the efficiency of conversion of ingested food (ECI = larval mass gain / stem mass ingested), and the efficiency of conversion of absorbed food (ECD = larval mass gain / (stem mass ingested – frass)). Percent mass gain was calculated as: [(mass gain in 24 h /initial mass) * 100] and the calculation of RGR utilized was as described by Waldbauer [89].

### Tissue Preparation and Microscopy Techniques

Tissue samples for analysis with light microscopy were fixed overnight in 50% (v/v) ethanol, 5% (v/v) glacial acetic acid and 10% (v/v) formaldehyde in water. The nuclei were imaged using a Leica Model DmIL inverted fluorescent microscope with integrated modulation contrast. Digital photographs were made using a Nikon Ds-Fi1 CCD camera attached to the microscope and connected to a computer with Nikon NIS-Elements imaging software (v 3.00) and Adobe CS5 Extended software (v 12.0.4x32).

### Statistical Analyses

With the exception of the percent weight gain data that was arcsine square root transformed, all data was square-root transformed prior to statistical analysis [90]. Analysis of variance (ANOVA) and Tukey HSD tests were used to make multiple comparisons among control, wound, and ECB samples. A Student’s *t*-test was used to make comparisons between two treatments. All statistical analyses were performed with JMP 4.0 (SAS Institute, Cary, N.C.).

## Supporting Information

Figure S1 **Previous ECB stem attack for 48 h subsequently supports enhanced ECB growth.** **A**, Average (*n* = 18, + SEM) percent mass gain of 5^th^ instar ECB larvae over a 24 h period on control (C) and larval-conditioned stem tissues (LCT). **B**, Average (*n* = 3, + SEM) total protein extracted from control (C), wounded (W), and larval-conditioned stem tissue (LCT) after 24 h. **C**, Average (*n* = 3, + SEM) glucose, fructose and **D**, free stearic (18: 0), oleic (18:1), and linolenic acid (18:3) in control (white bars), wound (grey bars), and larval-conditioned tissue (LCT, black bars) after 48 h. Significant differences are indicated by asterisk (Student’s *t*-test, *P* < 0.05), not significantly different (n.s.d) indicates *P* > 0.05 for ANOVAs.
(TIF)

Figure S2 iTRAQ experimental design illustrating the comparison of proteins extracted from untreated control and ECB-LCT tissue after 48 h.
(TIF)

Table S1 Microarray analysis of gene expression comparing untreated, control stem tissue to 48 h ECB damaged stem tissue.
(XLSX)

Table S2 Categories of genes that were differentially regulated in response to 48 h ECB feeding.
(XLSX)

Table S3 List of 171 proteins identified by LC-ESI MS/MS analysis when comparing 48 hr control and ECB-damaged maize stem tissue.
(XLSX)

Table S4
