# Supplementary figures and images for: European Corn Borer (Ostrinia nubilalis) Induced Responses Enhance Susceptibility in Maize
Source: PLoS One. 2013 Sep 2;8(9):e73394. doi: 10.1371/journal.pone.0073394 (PMC3759431; doi:10.1371/journal.pone.0073394)

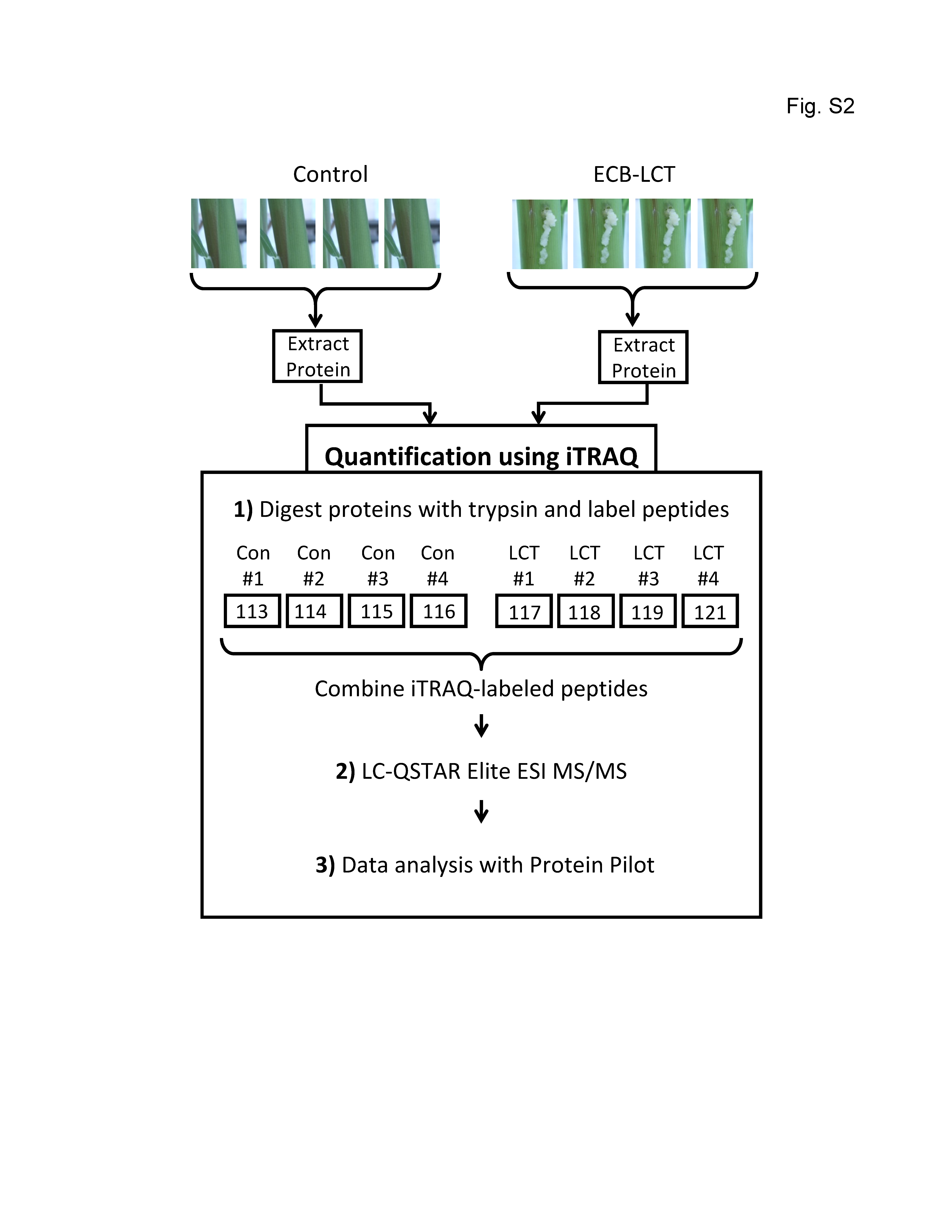

Supplement: Figure S2 — iTRAQ experimental design illustrating the comparison of proteins extracted from untreated control and ECB-LCT tissue after 48 h. (TIF) [file pone.0073394.s002.tif]
