# Supplementary material for: European Corn Borer (Ostrinia nubilalis) Induced Responses Enhance Susceptibility in Maize
Source: PLoS One. 2013 Sep 2;8(9):e73394. doi: 10.1371/journal.pone.0073394 (PMC3759431; doi:10.1371/journal.pone.0073394)
Supplement: Table S4 [file pone.0073394.s006.pdf]

Supplemental Table IV. *Primer sequences used for qRT-PCR analyses*

Symbol	NCBI Accession No.	Forward Primer	Reverse Primer	Amplicon Size (bp)
Pr10	NM_001137540	GTTTCTGCTACCTGTCTGACC	TGTACCTGATGCACTGCTTG	93
Sh-1	NM_001111941	ACTGATAGCACTCTTTTCCAGG	GGGTGCATACTTCTCCTTGTC	115
Lox1	DQ335760	TCGTCGCACTAGTTGTTTGC	TCCTCACGTAGCGACTTCTG	112
Lox2	DQ335761	TCCTGGAAGGCTACCTCCTC	ACCATCTGTAGCGGGAACTG	132
Mpi	EU972028	GGTGATTCTGGCGGACAAG	ACACGGTCGAACCCAAAG	90
Abp20	NM_001137400	CACTTCCAGTACAGCCACG	CACCTTGTTCACGACCTCC	132
GH3	NM_001158280	AGATGCCCGTAATGAACCTG	GTTCTTGAAGTGGTTGCTCTTG	149
Enod93-1	NM_001138337	TGAACATCGATCTGCTACTGC	GACCACATAAGCGTACTAGGG	143
Enod93-2	NM_001153581	GCACCTCAAGAACACCTCC	ACTGACGAACTGAAAGAGGC	116
Gpc3/4	U45856	GGTGTCAACGAGAAGGAGTAC	AGTGATGGCATGAACAGTGG	147
ETIF	NM_001157877	AGCCAAGAGAAGTGGTGC	TTCCTTAAGATCCACCTTCAGAAG	149
HD	NM_001111607	CCTGGTGTTCAAATGCAAGAG	TGTCATTGTCACCTTCATAGTACC	148
RpL17	AF034948	CTGAGGGTCCACTTCAAGAAC	CAACACCTCCACAGTATCTCC	139
